# Chronic kidney failure following lancehead bite envenoming: a clinical report from the Amazon region

**DOI:** 10.1590/1678-9199-JVATITD-2020-0083

**Published:** 2020-12-14

**Authors:** Manuela B. Pucca, Michelle V. S. Franco, Jilvando M. Medeiros, Isadora S. Oliveira, Shirin Ahmadi, Felipe A. Cerni, Umberto Zottich, Bruna K. Bassoli, Wuelton M. Monteiro, Andreas H. Laustsen

**Affiliations:** 1 Medical School, Federal University of Roraima, Boa Vista, RR, Brazil.; 2 Department of BioMolecular Sciences, School of Pharmaceutical Sciences of Ribeirão Preto, University of São Paulo (USP), Ribeirão Preto, SP, Brazil.; 3 Department of Biotechnology and Biomedicine, Technical University of Denmark, Kongens Lyngby, Denmark.; 4 School of Health Sciences, Amazonas State University, Manaus, AM, Brazil.; 5 Department of Teaching and Research, Dr. Heitor Vieira Dourado Tropical Medicine Foundation, Manaus, AM, Brazil.

**Keywords:** Bothrops, Snakebite, Long-term effects, Chronic kidney disease, Renal failure

## Abstract

**Background:**

Snakebite envenoming can be a life-threatening condition, for which emergency care is essential. The *Bothrops* (lancehead) genus is responsible for most snakebite-related deaths and permanent loss of function in human victims in Latin America. *Bothrops* spp. venom is a complex mixture of different proteins that are known to cause local necrosis, coagulopathy, and acute kidney injury. However, the long-term effects of these viper envenomings have remained largely understudied.

**Case presentation:**

Here, we present a case report of a 46-years old female patient from Las Claritas, Venezuela, who was envenomed by a snake from the *Bothrops* genus. The patient was followed for a 10-year period, during which she presented oliguric renal failure, culminating in kidney failure 60 months after the envenoming.

**Conclusion:**

In Latin America, especially in Brazil, where there is a high prevalence of *Bothrops* envenoming, it may be relevant to establish long-term outpatient programs. This would reduce late adverse events, such as chronic kidney disease, and optimize public financial resources by avoiding hemodialysis and consequently kidney transplantation.

## Background

The World Health Organization categorizes snakebite envenoming as a category A Neglected Tropical Disease [1], with more than one million cases occurring worldwide each year, and around 30,000 cases taking place in the tropical regions of Latin America [2]. In the Brazilian Amazon, the case fatality rate of snakebites has been estimated to be 0.51%, which is 10 times higher than the estimated global average [2,3]. *Bothrops*, *Crotalus*, *Lachesis,* and *Micrurus* are the medically most important snake genera in Brazil, with the *Bothrops* genus (lancehead pitvipers) being responsible for the majority of bites (90%) and snakebite related deaths (0.3%) within the Brazilian Amazon [3,4]. Among the 30 species of *Bothrops* snakes that are found in Brazil [5,6], *Bothrops atrox* (common lancehead, Amazonian jararaca) is considered to be the medically most important species due to its high level of adaptation and wide distribution in both rainforests and populated areas [7,8]. 

Venomics studies have revealed that the venom of*B. atrox* contains a limited number of protein families, including snake venom metalloproteinases (SVMP), L-amino acid oxidases (LAAO), C-type lectin-like (CTL) proteins, and snake venom serine proteinases (SVSP) [9-11]. Together, these protein families comprise almost 90% of the venom and are responsible for the reported clinical manifestations of *B. atrox* envenomings, such as coagulation disturbances, blood pressure alterations, and acute kidney injury (AKI), with the latter being very common in victims bitten by *B. atrox* species [12-14]. Indeed, AKI is known to be the main systemic complication and cause of death among the patients who survive the early effects of the venom [4,15]. However, the mechanisms behind the development of AKI are not completely elucidated. It is, however, known that isolated CTLs from the venom of *B. atrox* are capable of altering renal function, although it seems that different toxins in the whole venom must act synergistically to induce the AKI [16-18].

Considering the high chance of developing AKI among the victims bitten by a snake from the *Bothrops* genus, adequate hydration must be undertaken as an immediate treatment to protect the patient’s kidneys [4,19]. Administration of antivenom no later than six hours from the envenoming is, however, the most important treatment for preventing AKI [20]. Late medical assistance is common in remote areas of the Amazon region, and lack of proper hospital care often poses as a significant challenge. Moreover, those who seek treatment from the healthcare centers are rarely monitored after they leave the hospital. Therefore, limited knowledge exists on the long-term effects of snakebites in surviving victims. Here, we report a chronic kidney disease over 10 years following an incident of *Bothrops* spp*.* envenoming and provide case information on the long-term effects of lancehead venom on kidney function.

## Case presentation

**Figure 1.**
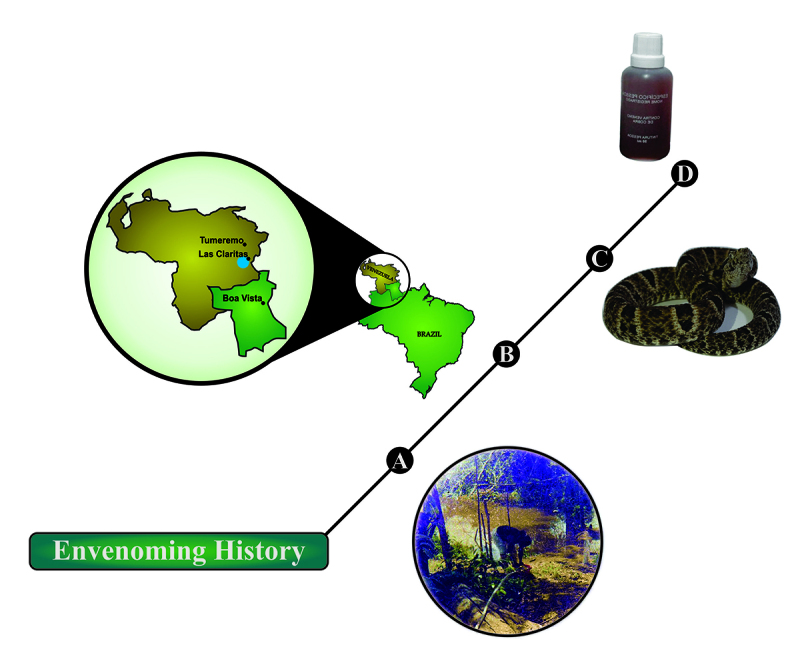
Envenoming history of the patient. **(A)** Patient working in a gold mine (photo provided by the patient). **(B)** Geographical location of the snakebite. Right panel shows Roraima, the northernmost state of Brazil, bordering Venezuela. Left panel shows the cities of Tumereno and Las Claritas in Venezula; Boa Vista, the capital of Roraima state; and in blue the predicted region of the snakebite. **(C)** *Bothrops atrox* snake, the most abundant pit viper species found in Roraima state and bordering areas in Venezuela (photo kindly provided by Anderson Maciel Rocha). **(D)** ‘Específico Pessoa’, an herbal mixture produced using an Amazon root extract.

On April 5^th^, 2010, approximately at 18:00, a 46-year-old Brazilian woman was bitten by a *Bothrops* spp. snake in the right lower dorsum of the foot (through her strap sandal), while she was working in a clandestine gold mine near Las Claritas (Fig. 1A), located in the state of Bolívar, Venezuela (Fig. 1B). The victim’s husband killed the snake, a dark yellow animal with black triangles, which was supposedly a small lancehead, locally known as “jararaca” (Fig. 1C).

The patient reported to instantly feel severe pain in the bitten foot, and as a traditional behavior of the goldminers, she quickly orally took 50 mL of ‘Específico Pessoa’ (Fig. 1D). The ‘Específico Pessoa’ is a herbal mixture produced from an Amazon root extract, popularly called ‘snake root’. According to the traditional culture, this extract can be used to treat envenomings caused by snakes, scorpions, and spiders. Despite the fact that it has not been approved by any pharmaceutical regulatory agency, such as the Brazilian ANVISA, ‘Específico Pessoa’ is very popular among the Amazonian population and is often used after snakebite envenomings [21-23]. The victim had to spend the night in a hut close to the gold mine with her right leg upwards, since the accident happened in the evening and the goldmine was located in an isolated area. 

The next day, she was carried in a hammock to the river, crossed the river by canoe, and reached the nearest healthcare center in Las Claritas. The patient arrived at Las Claritas hospital on April 6^th^, 2010, at 17:00 presenting pain, edema, paresthesia, sweating, and hematuria. Since antivenom was not available at the Las Claritas healthcare center at the time, the patient only received intravenous hydration, penicillin, and corticosteroids. As the clinical conditions of the envenoming had worsened over the last 72 hours, the victim was referred to the emergency unit of the Hospital Geral de Roraima (HGR), Boa Vista, Roraima, Brazil.

The patient arrived in HGR on April 8^th^, 2010, at 12:00. Upon arrival, she was afebrile, with a blood pressure of 150/80 mmHg, and without cardiorespiratory alterations. The patient denied having any previous comorbidities, especially diabetes, arterial hypertension or metal intoxication (*e.g.* mercury). Foot edema and necrosis and phlogistic signs surrounding the bite site were seen in the initial medical examination and the laboratory exams exhibited normal numbers of blood cells, liver enzymes, and creatinine kinase levels. Regarding kidney function, serum and urine analyses revealed acute renal failure: Serum creatinine of 5.3 mg/dL (reference range: 0.7 - 1.4 mg/dL) and serum urea of 169 mg/dL (reference range: 15 - 40 mg/dL) (Table 1, day 3). Urine analysis from day 3 showed hematuria and proteinuria.

**Table 1.** Laboratory analysis (2010 and 2012).

Laboratory serum analysis	Days after snakebite					Reference range^#^
	3	5	6	8	~720	
Glucose	124*	-	108*	-	82	60.0-99.0 mg/dL
Creatinine	5.3*	5.3*	4.1*	2.5*	1.5*	0.7-1.4 mg/dL
Urea	169*	185*	133*	88*	65*	15-40 mg/dL
Erythrocytes	4,400	4,900	4,130	-	4,960	4,000-5,200 10^3^/µL
Hemoglobin (Hb1_C_)	12.8*	11.5*	11.5*	-	14.2	13.5-18.0 g/dL
Hematocrit	39.2*	36.6*	37.6*	-	41	40.0-50.0%
Leucocytes	12,000*	7,800	9,400	-	9,390	4,000-10,000 cells /µL
Platelets	79,000*	74,000*	82,000*	-	115,000	150,000-400,000 /µL
Na^+^	-	135.7	134.8*	-	-	135.0-145.0 mmol/L
Cl^-^	-	103.9	98.3	-	-	98.0-107.0 mmol/L
Ca^2+^	-	1.02	1.18	-	-	1.17-1.32 mmol/L
K^+^	-	4.46	4.06	-	-	3.5-5.1 mmol/L

*Indicates that results were out of the reference range. ^#^Reference values are from the *Laboratório Central de Roraima* (LACEM - HGR), Boa Vista, Roraima, Brazil.

At the border between Roraima and Venezuela, where the patient comes from, the most common snake is the viper *B. atrox* [6,24]. Besides the patient who confirmed that the snake was a lancehead, clinical features were in agreement with viper venom-induced effects, as most literature shows that vipers can cause acute kidney injury and coagulopathy [4,5,7]. 

The patient was treated with symptomatic medication, proper hydration, and wound care, but she did not receive *Bothrops* antivenom, as it was evaluated by the treating personnel that she had arrived too late to the healthcare center for antivenom to be effective. The clinical basis for this was the fact that the recommended maximum time between envenoming and antivenom administration for getting the best neutralization effect is 6 to 24 h [25-27]. It is, however, likely that the patient would have benefitted from receiving antivenom beyond the recommended time interval. The patient was monitored continuously for 5 days and was discharged on day 8 after clinical improvement. The patient was not referred to a specialist or asked to return after discharged.

## How was the patient outcome?

**Figure 2.**
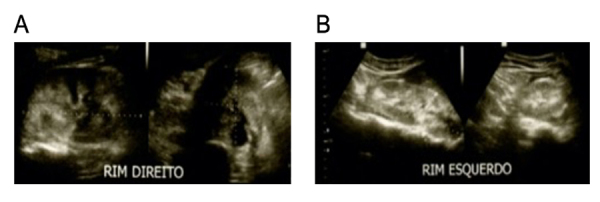
Ultrasonographic image from the **(A)** right and **(B)** left kidney (2012). Images demonstrate increased kidney size and normal parenchymal echogenicity. Kidney length, width, and thickness were 10.4 x 5.5 x 5.2 cm and 13.3 x 4.8 x 4.6 cm for right and left kidney, respectively. Renal volumes were 158 and 156 cm^3^ for the right and left kidney, respectively. The exam report suggested bilateral nephritis. The exam was performed on May 2^nd^, 2012.

After discharge from the hospital, the patient returned to her normal life and work activities and reported to feel well over the subsequent year. At the end of 2011, she started feeling pain in her back, she frequently observed that her feet and ankles were swollen, and her urine was foamy. She lost weight and easily felt fatigability, which she believed resulted from the excessive physical labor in the goldmine. Only in May 2012 (two years after the envenoming), she finally sought medical attention. Ultrasonography (Fig. 2) and laboratory tests (Table 1, ~720 days) confirmed that the patient suffered from chronic kidney disease (CKD). It was excluded that CKD was induced by diabetes or metal intoxication (*e.g.* mercury) based on glucose and liver enzymes ((-glutamyl transpeptidase, aspartate transaminase, and alanine transaminase) levels. Moreover, the patient had no family history of hypertension or kidney disease. The patient received prescription for unspecified ‘therapy’ and diet recommendation for the chronic kidney disease. For reference, it has been shown in previous studies that 41% of patients with kidney injury due to snakebite present CKD progression at a mean follow-up of 45 months [28].

In 2015 (five years after the snakebite incident), the patient returned to the hospital presenting severe kidney problems. Laboratory tests from 2015 revealed high creatinine, urea, calcium, potassium, and sodium serum levels, and the glomerular filtration rate (GFR) was estimated to be lower than 5 mL/min /1.73 m^2^, confirming that the patient had developed kidney failure. One more time it was excluded that other diseases could have induced the kidney failure (*e.g.* diabetes, hypertension, and metal intoxication). The patient, therefore, started renal replacement therapy.

## What long-term treatment did the patient receive following hospital discharge?

**Figure 3.**
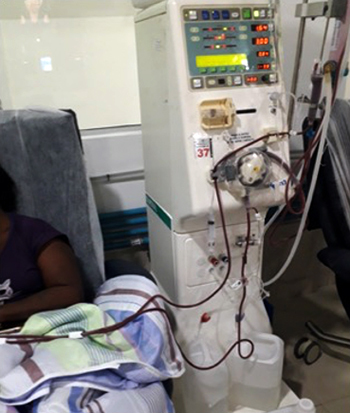
Patient connected to a hemodialysis machine at Clínica Renal de Roraima*.* The patient performs 3.5-hour hemodialysis cycles three times per week. Photo captured on January 7^th^, 2019.

**Figure 4.**
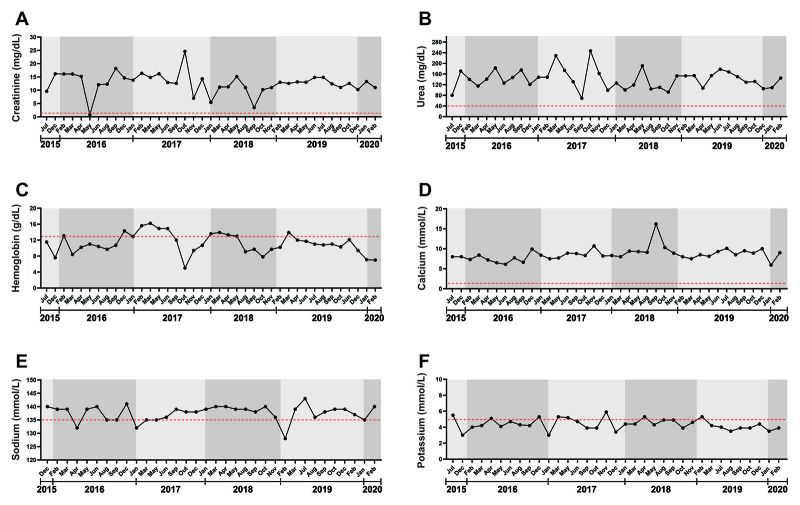
Laboratory analysis of the patient’s blood over the last five years (2015-2020). Serum levels of **(A)** creatinine, **(B)** urea, **(C)** hemoglobin, **(D)** calcium, **(E)** sodium, and **(F)** potassium. Red dotted lines show reference values, which highlight that the patient’s serum levels are higher than the reference range (**A**, **B**, and **D**), or vary between the normal and altered values (**C**, **E**, and **F**). Reference range according to *Laboratório Central de Roraima* (LACEM - HGR), Boa Vista, Roraima, Brazil: creatinine: 0.7-1.4 mg/dL; urea: 15-40 mg/dL; hemoglobin: 13.5-18.0 g/dL; calcium: 1.17-1.32 mmol/L; sodium: 135.0-145.0 mmol/L; potassium: 3.5-5.1 mmol/L.

During the last five years (2015-2020), the patient has attended hemodialysis sessions twice a week (Fig. 3), receiving IV erythropoietin and IV iron hydroxide. She also continuously receives clopidogrel, acetylsalicylic acid, nifedipine, methyldopa, losartan, cinacalcet, and sevelamer. However, her laboratory tests continue to show altered serum levels of creatinine, urea, ions (sodium, potassium, and calcium), and hemoglobin, out of the reference range (Fig. 4).

Moreover, since hemodialysis started, the patient passed through 13 minor surgical procedures to provide access for the needles that connect her blood circulation to the dialysis machine. In detail, she had ten surgeries for inserting central venous catheters (CVCs) and three surgeries for inserting native arteriovenous fistulae (AVFs).

These long-term consequences of the snakebite have prevented the patient from working since 2015, which significantly compromised her family’s income. In fact, for the time being, the patient's income is only based on a government benefit, which consists of a minimum financial support for disabled people who cannot provide for themselves. 

Renal transplantation is the best medical option for the victim’s end-stage chronic renal disease, and she has been on the Brazilian waitlist for a kidney transplant since 2015. 

## Discussion

All the four families of medically important venomous snakes, *i.e.* Elapidae, Viperidae, Hydrophiidae, and Colubridae, seem to be capable of inducing renal damages, including AKI, in their victims [19,29-32]. Global epidemiological data on snakebite-induced AKI is scarce, although AKI has been reported in 5-30% of snakebite envenoming cases, depending on the snake species and the severity of the envenoming [4,29,33]. It seems that AKI is predominantly caused by sea snakes in certain countries, such as Sri Lanka and Australia, and vipers in other parts of the world, including Latin America and South/South-East Asia [4,34-36]. 

The *Bothrops*, *Crotalus*, and *Daboia* genera of vipers are considered to be responsible for the majority of snakebite-induced AKI cases reported worldwide [4,37,38]. In Brazil, in cases where the victim sought medical attention at a healthcare center, and the perpetrating snake species was identiﬁed, *Bothrops* snakes were responsible for 90.5% of the all envenomings, 0.3% of snakebites death, and 1.6-38.5% of AKI [4]. These numbers are alarming, especially when considering the fact that due to underreporting of snakebites, the magnitude of the problem is likely to be much larger. This puts emphasis on the importance of immediate and effective treatment of the victims bitten by *Bothrops* snakes, as untreated AKI may lead to CKD. When CKD develops, this will usually require renal replacement therapy, *i.e.* dialysis, which is rarely available or affordable in resource-poor regions of the tropics. 

Although the pathophysiology of snakebite-induced AKI is poorly understood [7], different mechanisms, including hypotensive effects, myoglobinuria, direct action on the kidney, hemoglobinuria, disseminated intravascular coagulopathy (DIC) or thrombotic microangiopathy, and glomerular microthrombi deposit, have been attributed to AKI caused by snakebite envenomings [39]. While hypotension and myoglobinuria are unlikely to be important factors in the pathogenesis of renal injury after a *Bothrops* snakebite (these effects are mostly caused by rattlesnake envenomings) [4,32], SVMPs and PLA_2_s, that are abundantly found in the venom of *Bothrops* species (including *Bothrop atrox*), can cause direct nephrotoxicity in victims. SVMPs have a proteolytic effect on the extracellular matrix and can disrupt the cellular adhesion through degradation of the major components of the basal lamina [40]. PLA_2_s can cause membrane injury either via hydrolysis of membrane glycerophospholipids at the *sn*-2 site of these molecules or via non-catalytic membrane disruption, which may cause tubular necrosis, which is an important pathological mechanism of AKI [41,42]. Besides direct injuries, SVMPs and PLA_2_s indirectly damage the kidneys by inducing cytokines and inflammatory mediators, which in turn can lead to renal ischemia [29]. Hemoglobinuria, caused by intravascular hemolysis, is frequently reported after *Bothrops* envenomation and might also contribute to renal injury [36]. Unfortunately, the description of this case is based only on the information available in the patient's medical records, which is subject to the criterion adopted by the attending physician and local availability of diagnostic resources, which is limited in the location where the patient was treated. For example, the presence of DIC or thrombotic microangiopathy cannot be discarded in this patient, especially considering the occurrence of thrombocytopenia.

One of the main issues in regard to snakebite-induced AKI is that after an apparent recovery in victims, it may progress to CKD. There are very few studies that monitored snakebite-induced AKI among the victims over longer periods of time to evaluate the risk of AKI progressing to CKD [28,43-45]. The inherent limitations of these studies include a lack of information on renal function of patients prior to their envenoming incident, the identity of the perpetrating snake species, standardized follow-up programs, standardized methods for determining the severity of initial kidney injury, and differences in interventions made during the acute phase of envenoming. These limitations all make it difficult to determine the prevalence, predictors, and time scale of AKI progression to CKD [31]. However, according to some studies, it can be expected that 37-41% of snakebite victims develop CKD after 12-45 months from their snakebite incident [28,35]. Only about 5% of these patients are, however, likely to progress to end-stage renal disease [28]. Unfortunately, the victim of this report belonged to this patient population. By provision of regular follow-up and treatment for the patients, who have AKI and CKD, end-stage renal disease may be preventable in most cases. However, there will still be cases where renal failure is unavoidable, and where snakebite victims must thus start dialysis. 

The provision of hemodialysis following snakebites is a real challenge for low-income countries, such as Brazil, which are overburdened with patients requiring it. A recent study conducted in China has estimated that one hemodialysis session costs US$ 386.6 [46]. In Brazil, according to the Brazilian healthcare system (*Sistema Único de Saúde* - SUS), a hemodialysis procedure costs US$ ~40 per session and US$ ~7,500 per patient per year [47], which is, however, covered by the government. It must be noted, though, that these estimated costs do not include clinician salaries, machine maintenance, medication, or other medical care costs. For instance, depression, with a prevalence from 22.8% to 39.3%, is a very common psychological disease in patients who are dependent on hemodialysis, and this co-morbidity must be treated as well [48]. Renal transplantation is considered to be a cost-saving and preferable alternative for patients under hemodialysis, since a renal transplantation is estimated to cost US$ 10,279, which is at a similar cost level as one year of hemodialysis treatment [46]. However, due to scarcity of organ donations, it cannot be considered as an easily accessible alternative. 

## Conclusion

In Latin America, especially in Brazil, with a high prevalence of *Bothrops* snakebites, it may be relevant to establish follow-up programs for snakebite patients for an extended period of time to better understand and reduce the risk of long-term envenoming effects. It is also important to supply and distribute specific antivenoms in the regions most at risk of snakebite evenoming. Early treatment can prevent the progression of AKI to CKD by avoiding hemodialysis and consequently kidney transplantation.

### Abbreviations

AKI: acute kidney injury; ANVISA: Agência Nacional de Vigilância Sanitária (Brazilian Health Regulatory Agency); AVF: arteriovenous fistula; CKD: chronic kidney disease; CTL: C-type lectin-like; CVC: central venous catheter; GFR: glomerular filtration rate; HGR: Hospital Geral de Roraima; LAAO: L-amino acid oxidase; PLA_2_: phospholipase A_2_; SVMP: snake venom metalloproteinase; SVSP: snake venom serine proteinase.
